# Nano-Selenium-Mediated Alleviation of Chromium Toxicity and Selenium Biofortification of *Ipomoea aquatica* Forssk. in Cyclic Hydroponic System

**DOI:** 10.3390/plants15152279

**Published:** 2026-07-25

**Authors:** Mingxuan Wang, Yunting Wang, Shuangqi Yue, Fengyue Qin, Menglu Dong, Wenxin Wang, Xinyu Shan, Waqas Ahmed, Sajid Mehmood, Weidong Li

**Affiliations:** 1 International Joint Research Center for Coral Reef Ecology of Hainan Province, School of Ecology, Hainan University, Haikou 570228, China; 2 Center for Eco-Environment Restoration of Hainan Province, School of Ecology, Hainan University, Haikou 570228, China; 3 College of the Environment & Ecology, Xiamen University, Xiamen 361102, China

**Keywords:** selenium nanoparticles, cyclic hydroponics, antioxidant enzymes, biofortification, sodium selenite, oxidative stress

## Abstract

Chromium (Cr) contamination poses a serious threat to agricultural productivity and food safety, yet effective strategies for mitigating Cr toxicity under practical cultivation conditions remain limited. In this study, green-synthesized selenium nanoparticles (SeNPs), prepared from banana peel extract (average particle size: 112.9 nm), were evaluated against conventional sodium selenite (Na_2_SeO_3_) for alleviating Cr(VI) toxicity in *Ipomoea aquatica* Forssk. grown in a dynamic cyclic hydroponic system. Plants were exposed to 20 mg L^−1^ Cr(VI) and treated with SeNPs or Na_2_SeO_3_ at 1 and 10 mg L^−1^, respectively, for a duration of one week. Green-synthesized SeNPs exhibited excellent colloidal stability (zeta potential: −35.5 mV) and an amorphous spherical morphology. Relative to the Cr-only treatment, 10 mg L^−1^ SeNPs almost completely restored plant biomass and root growth, decreased shoot Cr accumulation by 62.2%, and recovered total chlorophyll content to 94.8% of the control level. By comparison, 10 mg L^−1^ Na_2_SeO_3_ restored total chlorophyll to 74.2% of the control and reduced shoot Cr accumulation by 50.6%. SeNPs also elicited a stronger antioxidant response, markedly increasing SOD, POD, and CAT activities while significantly lowering H_2_O_2_, MDA, and proline levels, demonstrating a greater capacity than selenite to alleviate Cr-induced oxidative damage. SeNPs also restored the uptake of essential mineral nutrients (N, P, K, Ca, and Mg), improved soluble sugar and protein contents, and promoted selenium biofortification in edible shoots. Although both selenium forms alleviated Cr-induced growth inhibition and oxidative damage, SeNPs consistently outperformed Na_2_SeO_3_ owing to their higher bioavailability and sustained physiological activity. Overall, this study demonstrates that green-synthesized SeNPs provide an efficient and sustainable strategy for reducing Cr accumulation while simultaneously improving crop growth, antioxidant capacity, and nutritional quality under agriculturally relevant hydroponic conditions, highlighting their potential for safer food production and agricultural waste valorization.

## 1. Introduction

The escalating industrialization of modern society has precipitated a severe global environmental challenge: heavy metal contamination [1]. Widespread industrial activities, including leather tanning, electroplating, and pigment manufacturing, coupled with the intensive use of agrochemicals, have led to the significant release of toxic heavy metals such as chromium (Cr) into soil and aquatic ecosystems [1]. Unlike organic pollutants, these metals are non-biodegradable, allowing them to persist in the environment and bioaccumulate in the food chain [2]. Among them, hexavalent chromium (Cr(VI)) is exceptionally hazardous and is classified as a Group 1 human carcinogen by the International Agency for Research on Cancer (IARC) due to its potent genotoxic and carcinogenic properties [3].

In agricultural systems, Chromium (Cr) toxicity poses a serious challenge by disrupting key plant physiological processes, including seed germination, root development, nutrient uptake, and photosynthetic efficiency [4]. As a persistent and non-biodegradable heavy metal, Cr contamination is difficult to remove from environmental matrices such as soil and water [4]. Consequently, elevated heavy metal concentrations in agricultural soils have become a global concern due to their adverse impacts on ecosystem stability and human health [3]. A primary driver of this phytotoxicity is the induction of oxidative stress, caused by an overproduction of reactive oxygen species (ROS) that leads to lipid peroxidation, cell membrane damage, and the inactivation of key metabolic enzymes [5,6]. While plants possess an innate antioxidant defense system—comprising enzymes like superoxide dismutase (SOD), catalase (CAT), and peroxidase (POD)—this system is often overwhelmed by high levels of Cr exposure [7]. Leafy vegetables such as water spinach (*Ipomoea aquatica* Forssk.) are widely cultivated and consumed throughout Asia because of their rapid growth, high biomass production, and excellent nutritional value. As an important leafy vegetable, *I. aquatica* is rich in dietary fiber, vitamins, carotenoids, and essential mineral nutrients, including calcium and potassium, making it an important component of a healthy diet. Owing to its fast growth, high productivity, and adaptability to hydroponic cultivation, it is frequently used as a model vegetable crop in controlled cultivation studies. However, *I. aquatica* also has a strong capacity to accumulate heavy metals, particularly chromium (Cr), in its edible tissues [5,8]. Even relatively low concentrations of Cr(VI) can significantly inhibit plant growth, impair physiological and biochemical processes, and increase the risk of heavy metal entry into the food chain. This high accumulation potential raises serious food safety concerns in regions where *I. aquatica* is widely consumed, highlighting the need for effective strategies to reduce Cr uptake while maintaining crop productivity and nutritional quality [6].

To counteract heavy metal-induced phytotoxicity, exogenous ameliorants such as Se have gained attention due to their ability to enhance antioxidant defense and reduce ROS damage, thereby improving plant tolerance and yield [9,10]. However, traditional inorganic forms like sodium selenite (Na_2_SeO_3_) have a narrow safety margin and may cause phytotoxicity at slightly elevated concentrations, particularly in controlled systems such as hydroponics. Nanotechnology offers a promising alternative, as selenium nanoparticles (SeNPs) exhibit higher bioavailability, stronger antioxidant capacity, and lower toxicity than inorganic Se [11,12]. Moreover, green synthesis using biological materials such as banana peels-rich in polyphenols and polysaccharides-provides an eco-friendly and cost-effective approach for SeNP production while promoting agricultural waste valorization and circular economy principles [13].

To comprehensively evaluate chromium (Cr)-induced phytotoxicity and the protective effects of selenium, this study employed a suite of physiological, biochemical, and elemental indicators representing key aspects of plant stress responses. Growth traits and chlorophyll content were assessed to evaluate plant development and photosynthetic performance, while antioxidant enzymes (superoxide dismutase, peroxidase, and catalase), together with hydrogen peroxide, malondialdehyde, and proline, were analyzed to characterize oxidative damage, membrane integrity, osmotic adjustment, and antioxidant defense. Soluble sugars and soluble proteins were determined as indicators of primary metabolism and stress adaptation, whereas the accumulation of Cr, Se, and essential mineral nutrients (N, P, K, Ca, and Mg) in roots and shoots was measured to elucidate metal uptake, translocation, nutrient homeostasis, and selenium biofortification. Collectively, these complementary parameters provide a comprehensive framework for elucidating the physiological mechanisms by which selenium nanoparticles alleviate Cr toxicity and improve plant performance under hydroponic conditions.

Despite previous studies [14,15,16] reporting that SeNPs enhance antioxidant enzymes (SOD, POD, CAT) and reduce MDA, H_2_O_2_, and Cr accumulation under static conditions, our study confirms these mechanisms in a dynamic recirculating hydroponic system. This system better simulates real cultivation conditions by continuously altering nutrient flow and nanoparticle behavior, thereby providing new evidence for the stability and practical applicability of SeNP-mediated stress tolerance in realistic agricultural environments. Therefore, this study was designed to evaluate and compare the efficacy of green-synthesized SeNPs and sodium selenite in mitigating Cr stress in *I. aquatica* within a dynamic, cyclic hydroponic system. Our objectives were to: (1) synthesize and comprehensively characterize SeNPs derived from banana peel extract; (2) evaluate and compare the regulatory effects of green-synthesized SeNPs and conventional sodium selenite on plant growth, physiological traits, and antioxidant responses of *I. aquatica* subjected to hexavalent chromium stress in a dynamic circulating hydroponic system; and (3) elucidate the internal mitigation mechanisms by analyzing the uptake, translocation, and accumulation of chromium in plants. This research seeks to bridge the gap between static laboratory incubation and practical agricultural production, thereby providing a scientific theoretical foundation for sustainable strategies aimed at safeguarding crop yield and food safety in chromium-contaminated environments.

## 2. Materials and Methods

### 2.1. Production of Nano-Selenium

SeNPs were synthesized through a green reduction approach using banana peel extract as both the reducing and stabilizing agent. Banana peel was selected because it is an abundant, inexpensive agricultural by-product that is often discarded as waste, making it an attractive and sustainable feedstock for green nanoparticle synthesis. In addition, banana peels are rich in bioactive phytochemicals, including polyphenols, flavonoids, and polysaccharides, which can efficiently reduce selenium ions and simultaneously stabilize the resulting nanoparticles [17]. These biomolecules adsorb onto the nanoparticle surface through functional groups such as hydroxyl, carboxyl, and carbonyl moieties, forming a negatively charged capping layer that enhances colloidal stability and helps prevent nanoparticle aggregation [9]. Thus, the use of banana peel extract not only provides an environmentally friendly synthesis route but also promotes agricultural waste valorization in line with circular economy principles. Fresh banana peels were cut into small pieces, washed thoroughly with deionized water to remove surface impurities, and dried in an oven at 80 °C for 24 h. The dried material was ground and passed through an 80-mesh sieve to obtain uniform banana peel powder. Subsequently, 10 g of powder was mixed with 100 mL of deionized water and extracted at 60 °C under constant stirring (200 rpm) for 1.5 h. After cooling to room temperature, the mixture was centrifuged for 15 min, and the supernatant was vacuum filtered through a 0.45 μm membrane to obtain a clear banana peel extract.

Sodium selenite (Na_2_SeO_3_) was used as the selenium precursor and was purchased from Liaoning Quan Rui Reagent Co., Ltd. (Liaoyang, Liaoning, China). A 0.1mol L^−1^ Na_2_SeO_3_ solution was prepared by dissolving 1.73 g of Na_2_SeO_3_ in 100 mL of deionized water. The banana peel extract was then added to the selenium solution at a volume ratio of 1:2 and the mixture was stirred at 60 °C and 200 rpm for 30 min. Ascorbic acid was subsequently introduced at a molar ratio of 3:1 relative to selenium. The reaction mixture was maintained at 60 °C in the dark overnight to facilitate nanoparticle formation. The appearance of a brick-red color indicated the successful formation of SeNPs. The resulting suspension was centrifuged to collect the precipitate, which was washed twice with deionized water and twice with absolute ethanol to remove residual reactants and soluble impurities. Finally, the purified product was freeze-dried overnight to obtain nano-selenium powder for subsequent experiments.

### 2.2. Materials Characterization

The physicochemical properties of the synthesized selenium nanoparticles (SeNPs) were comprehensively characterized using multiple analytical techniques. Fourier-transform infrared (FTIR) spectra were recorded using a Nicolet iS50 FTIR spectrometer (Thermo Scientific, Waltham, MA, USA) over the range of 400–4000 cm^−1^ to identify functional groups and confirm the presence of biomolecules involved in nanoparticle reduction and stabilization. The crystalline structure of the SeNPs was analyzed using an X-ray diffractometer (SmartLab, Rigaku Corporation, Tokyo, Japan) within a 2θ range of 10–80°, with a scanning time of 10 min, to determine their phase composition and crystallinity. Particle-size distribution and surface charge (zeta potential) were measured by dynamic light scattering (DLS) using a Zetasizer Nano ZS instrument (Malvern Panalytical Ltd., Malvern, UK), providing information on nanoparticle size, dispersion, and colloidal stability in suspension.

### 2.3. Hydroponic System Configuration

**Figure 1:**
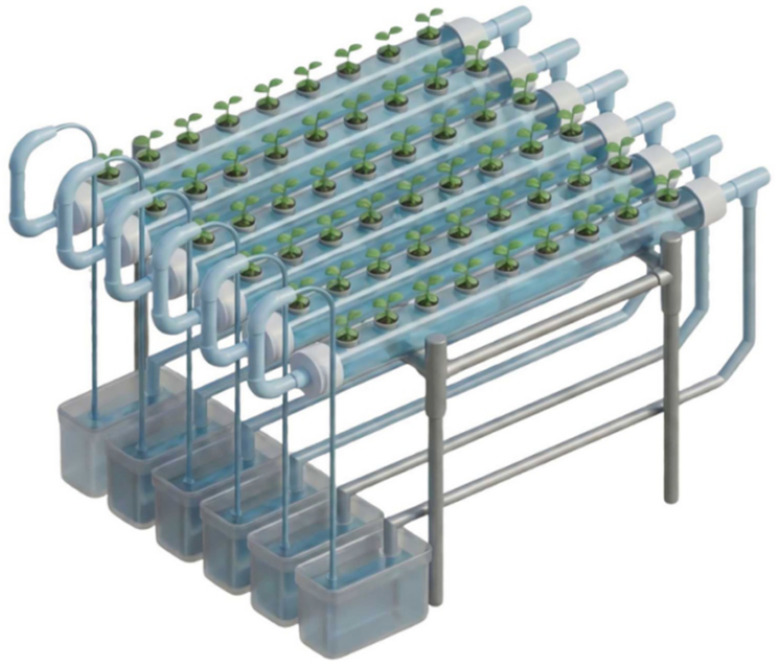
Schematic illustration of the dynamic cyclic hydroponic system used for cultivating *I. aquatica* under controlled chromium Cr(VI) stress.

To simulate realistic agricultural production conditions, a recirculating hydroponic system constructed from polyvinyl chloride (PVC) pipes was designed (Figure 1). The system consisted of six independent cultivation channels, each containing nine planting sites and an independent closed-loop nutrient circulation unit, allowing six treatment groups to be conducted simultaneously.

Each circulation unit was equipped with a water pump (16 W; flow rate 800 L h^−1^) to ensure continuous nutrient delivery to plant roots. Nutrient solution was pumped from the storage reservoir into the upper inlet of the pipeline and distributed to the plant roots before returning to the reservoir via a gravity-driven drainage outlet. This closed-loop configuration ensured efficient nutrient supply while minimizing volatilization and loss of contaminants.

The recirculating hydroponic system in the present study served as a controlled experimental platform for evaluating the remediation efficiency of SeNPs under Cr stress, thereby providing practical guidance for the potential application of this amendment to improve crop productivity and food safety under heavy metal contamination scenarios [18].

### 2.4. Plant Cultivation

*I. aquatica*, a three-pronged cultivar with strong disease resistance and high yield potential, was used in this study. Seeds were surface-sterilized in 5% sodium hypochlorite solution for 5 min and subsequently rinsed five times with ultrapure water to remove residual disinfectant. The sterilized seeds were soaked in warm water for 30 min and evenly distributed in substrate-free seedling trays for germination under shaded conditions at room temperature. During the 5-day germination period, humidity was maintained through regular water spraying.

Seedlings reaching approximately 7 cm in height were transferred to a recirculating hydroponic system. Growth conditions were maintained at day/night temperatures of 25/20 °C with a relative humidity of 75%. Plants were initially grown in 50% Hoagland nutrient solution for 5 days, followed by full-strength Hoagland solution, which was renewed every five days. Prior to treatment application, plants were acclimated in the nutrient solution for one month.

### 2.5. Experimental Design

The experiment consisted of six treatments: control (CK), chromium treatment (Cr), and four selenium treatments. The CK treatment contained Hoagland nutrient solution only, whereas the Cr treatment contained Hoagland solution supplemented with 20 mg L^−1^ Cr(VI). The solution is prepared by dissolving analytically pure potassium dichromate (K_2_Cr_2_O_7_) in deionized water according to a specific ratio. Based on previous studies [19], selenium was applied to the Cr treatment as either selenium nanoparticles (SeNPs) or sodium selenite (Na_2_SeO_3_) at two concentrations: A1 (10 mg L^−1^ SeNPs), A2 (1 mg L^−1^ SeNPs), B1 (10 mg L^−1^ Na_2_SeO_3_), and B2 (1 mg L^−1^ Na_2_SeO_3_). Each treatment consisted of an independent 5-L recirculating hydroponic system and was conducted with three independent biological replicates, resulting in a total of 18 experimental units. Each replicate contained nine plants grown under identical environmental conditions.

### 2.6. Sample Collection

After seven days of treatment, plants were harvested and separated into shoots and roots using sterilized scissors. Samples were thoroughly rinsed with ultrapure water to remove surface contaminants. Plant height and fresh biomass were recorded immediately after harvest. Samples were subsequently stored at −80 for further biochemical and physiological analyses.

### 2.7. Analytical Methods

#### 2.7.1. Pigment Content Analysis

Chlorophyll a, chlorophyll b, and total chlorophyll contents were determined using a Plant Chlorophyll Assay Kit (G0601W, Suzhou Geris Biotechnology Co., Ltd., Suzhou, China) following the manufacturer’s instructions. The assay is based on solvent extraction of chlorophyll pigments and spectrophotometric measurement according to the method of Arnon [20]. Absorbance was measured using a Thermo Scientifi^TM^ Multiska^TM^ SkyHigh Microplate Spectrophotometer (Thermo Fisher Scientific Inc., Waltham, MA, USA). Chlorophyll concentrations were calculated using the following equations:

$$Chlorophyll\;a\;\left(mg\;g^{-1}\right)=12.7A_{663}-2.69A_{645}$$

$$Chlorophyll\;b\;\left(mg\;g^{-1}\right)=22.9A_{645}-4.68A_{663}$$

$$Total\;chlorophyll\;\left(mg\;g^{-1}\right)=20.2A_{645}+8.02A_{663}$$

where *A* represents absorbance at the corresponding wavelength.

#### 2.7.2. Oxidative Stress Assessment

The activities of superoxide dismutase (SOD), peroxidase (POD), and catalase (CAT) were determined using commercial assay kits (G0101W, G0107W, and G0105W, respectively; Suzhou Geris Biotechnology Co., Ltd., Suzhou, China) according to the manufacturer’s instructions. SOD activity was determined using the WST-8 method based on inhibition of nitro blue tetrazolium reduction, where one unit (U) of SOD activity was defined as the amount of enzyme causing 50% inhibition of the reaction. POD activity was determined from the oxidation rate of guaiacol monitored at 470 nm and expressed as the increase in absorbance per minute. CAT activity was determined from the decomposition rate of H_2_O_2_ by monitoring the decrease in absorbance at 240 nm.

Hydrogen peroxide (H_2_O_2_), malondialdehyde (MDA), and proline contents were determined using commercial colorimetric assay kits (G0168W, G0109W, and G0111W, respectively). MDA concentration was calculated according to the thiobarbituric acid (TBA) reaction using

$$MDA=6.45\;\left(A_{645}-A_{600}\right)-0.56A_{450}$$

H_2_O_2_ concentration was calculated from the standard calibration curve supplied with the assay kit, while proline concentration was determined from a standard curve generated using known proline standards.

Soluble protein and soluble sugar contents were determined using the Coomassie Brilliant Blue method [21] and the anthrone–sulfuric acid method [22], respectively, with commercial kits (G0417W and G0501W). Protein and sugar concentrations were calculated from calibration curves prepared using bovine serum albumin (BSA) and glucose standards, respectively. All absorbance measurements were performed using a Thermo Scientific^TM^ Multiskan^TM^ SkyHigh Microplate Spectrophotometer (Thermo Fisher Scientific Inc., Waltham, MA, USA). All spectrophotometric measurements were performed following the manufacturers’ instructions and established analytical procedures commonly used in plant stress physiology [23,24,25,26].

#### 2.7.3. Elemental Analysis

Dried plant samples were ground into fine powder, and approximately 0.2 g of each sample was digested with concentrated HNO_3_ using a microwave digestion system. Chromium concentrations were determined according to the Chinese National Food Safety Standard (GB 5009.268-2016) [27]. The concentrations of Cr, Se, P, K, Ca, and Mg were quantified using an Agilent 7900 inductively coupled plasma mass spectrometer (ICP-MS; Agilent Technologies, Santa Clara, CA, USA) with the internal standard method. Element concentrations were calculated from external calibration curves generated using certified standard solutions, and quality control was performed using reagent blanks and standard reference materials.

### 2.8. Statistical Analysis

All statistical analyses were performed using IBM SPSS Statistics 26.0 (IBM Corp., Armonk, NY, USA). Differences among treatments were evaluated by one-way analysis of variance (ANOVA), followed by Tukey’s honestly significant difference (HSD) test for multiple comparisons at a significance level of *p* < 0.05. Data are presented as the mean ± standard deviation (SD) of three biological replicates. Figures were prepared using Origin 2025b (OriginLab, Northampton, MA, USA), and the graphical abstract and mechanism illustration were created using BioRender (https://app.biorender.com).

## 3. Results and Discussion

### 3.1. Characterization of Green-Synthesized SeNPs

Comprehensive characterization was performed to identify the physicochemical properties of banana peel-derived SeNPs. Structural, morphological and surface chemical features govern the stability, reactivity and biofunctions of SeNPs in plants. Particle size, crystallinity and surface groups greatly affect their bioavailability and membrane interactions [28,29]. Benefiting from tiny size and large specific surface area, SeNPs exhibit strong catalytic and antioxidant capacity to mitigate heavy metal stress in plants [30,31]. Bioactive substances from banana peel serve as capping stabilizers during green synthesis and regulate nanoparticle bio-interactions [32]. Hence, full physicochemical characterization is necessary to clarify how SeNPs relieve Cr-caused phytotoxicity and oxidative damage.

Dynamic light scattering (DLS) analysis showed that the synthesized SeNPs exhibited an average hydrodynamic diameter of approximately 112.9 nm (Figure S1a). Such heterogeneity is commonly reported for nanoparticles synthesized via green routes, as plant extracts contain a complex mixture of polyphenols, proteins, carbohydrates, and other biomolecules that act simultaneously as reducing and stabilizing agents during nanoparticle formation [33]. These biomolecules can induce variable nucleation and growth rates during synthesis, resulting in particles of different sizes and degrees of aggregation. Similar particle size ranges (50–200 nm) have been reported for plant-mediated SeNPs synthesized using various botanical extracts, highlighting the influence of phytochemical composition on nanoparticle morphology and stability [28,34].

The presence of larger particles in the size distribution suggests partial aggregation of SeNPs, which may occur during synthesis or sample preparation prior to DLS analysis. Nanoparticle aggregation is frequently observed in green-synthesized systems due to intermolecular interactions among surface-bound biomolecules and the dynamic nature of colloidal suspensions [35]. Nevertheless, particle sizes within the nanometer scale are advantageous for biological applications because smaller particles exhibit higher surface area-to-volume ratios, which enhance their reactivity and interaction with biological membranes [36]. This property is particularly important in plant systems, where nanoscale materials can more readily interact with root surfaces and influence nutrient uptake and stress responses.

The colloidal stability of the synthesized SeNPs was further evaluated using zeta potential analysis. The average zeta potential value was −35.5 mV (Figure S1b), with two prominent peaks observed at −41.5 mV and −27.4 mV. Zeta potential values exceeding ±30 mV are generally indicative of stable colloidal systems due to strong electrostatic repulsion between nanoparticles that prevents aggregation [37]. The negative surface charge observed in this study suggests that the SeNPs possess strong colloidal stability, allowing them to remain well-dispersed in aqueous environments. This property is particularly critical in hydroponic systems, where nanoparticle dispersion directly influences their mobility, bioavailability, and interaction with plant roots.

The strong negative surface charge is likely associated with organic functional groups derived from banana peel extract that remain adsorbed on the nanoparticle surface. Such natural capping layers not only stabilize nanoparticles but can also improve their environmental compatibility and biological interactions [34]. Consequently, the observed nanoscale particle size and strong negative zeta potential indicate that the synthesized SeNPs possess favorable physicochemical properties for stable dispersion and potential uptake by plants in hydroponic environments.

**Figure 2:**
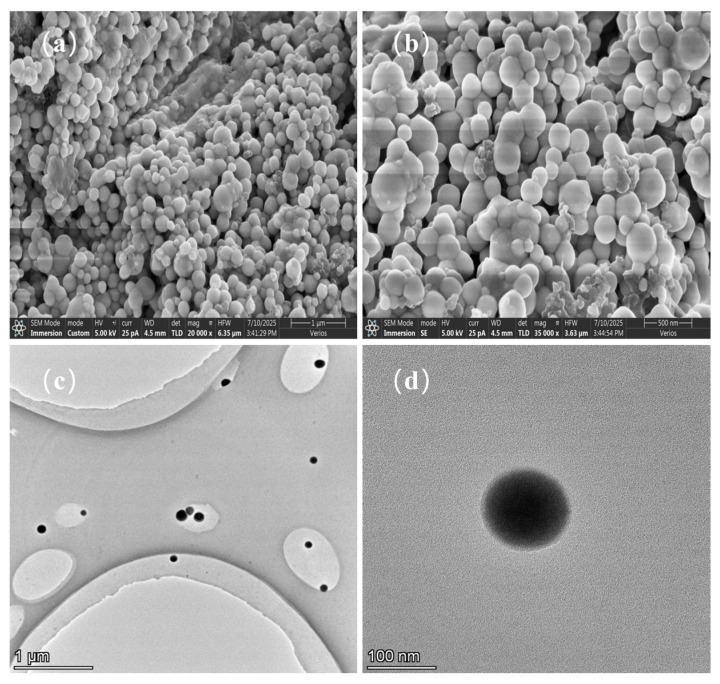
Morphological and structural characterization of green-synthesized selenium nanoparticles (SeNPs). (**a**,**b**) SEM images at different magnifications, and (**c**,**d**) TEM images.

Scanning electron microscopy (SEM) and transmission electron microscopy (TEM) were employed to investigate the morphology and internal structure of the synthesized SeNPs. SEM micrographs (Figure 2a,b) revealed that the particles were predominantly spherical to ellipsoidal in shape and relatively well dispersed, with an average particle size ranging from approximately 150 to 193 nm. This size range is generally consistent with the hydrodynamic diameter obtained from DLS analysis, although slightly larger values in SEM images may arise from particle aggregation during drying or differences between hydrodynamic and physical particle measurements [10]. The predominantly spherical morphology observed in this study is typical for biogenically synthesized SeNPs and is often attributed to the isotropic growth of nanoparticles during plant-mediated reduction processes [28]. Such morphology provides a relatively high surface-area-to-volume ratio, which can enhance surface reactivity and facilitate interactions with biological systems and environmental contaminants.

Transmission electron microscopy (TEM) images (Figure 2c,d) further confirmed the dense and solid internal structure of the nanoparticles and indicated good particle dispersion without significant agglomeration. The relatively uniform distribution observed in TEM supports the strong colloidal stability suggested by the zeta potential measurements. Similar nanoscale spherical SeNP structures have been widely reported in green synthesis studies and are considered favorable for biological applications due to their enhanced stability, cellular interaction, and antioxidant activity [38]. In plant systems, nanoscale selenium particles can interact more effectively with root surfaces and may improve stress tolerance by enhancing antioxidant defense mechanisms and regulating redox balance.

Energy-dispersive X-ray spectroscopy (EDS) analysis further verified the elemental composition of the nanoparticles. A prominent peak corresponding to selenium (Se) was observed in the spectrum (Figure S2), confirming the successful synthesis of SeNPs. In addition to selenium, minor peaks corresponding to carbon (C) and oxygen (O) were detected, which can be attributed to organic biomolecules derived from banana peel extract that remain adsorbed on the nanoparticle surface. These biomolecules likely originate from polyphenols, flavonoids, and polysaccharides present in the plant extract, which act as reducing and stabilizing agents during nanoparticle synthesis [39]. A trace amount of sodium (Na) was also detected, most likely originating from residual sodium selenite precursor or pH adjustment during synthesis.

Importantly, this organic coating should not be regarded merely as a residual byproduct but rather as a functional surface layer that enhances nanoparticle stability and environmental compatibility. Surface-bound biomolecules can prevent nanoparticle aggregation by steric and electrostatic stabilization while also improving their interaction with biological systems [40,41]. Moreover, such organic capping layers may facilitate the adsorption or complexation of heavy metal ions, thereby potentially contributing to detoxification processes and enhancing the protective effects of SeNPs under heavy metal stress conditions [42]. Consequently, the combined SEM, TEM, and EDS analyses confirm that the synthesized SeNPs possess well-defined morphology, stable dispersion, and a biomolecule-coated surface, which are advantageous properties for their potential application in mitigating chromium toxicity in plant systems.

**Figure 3:**
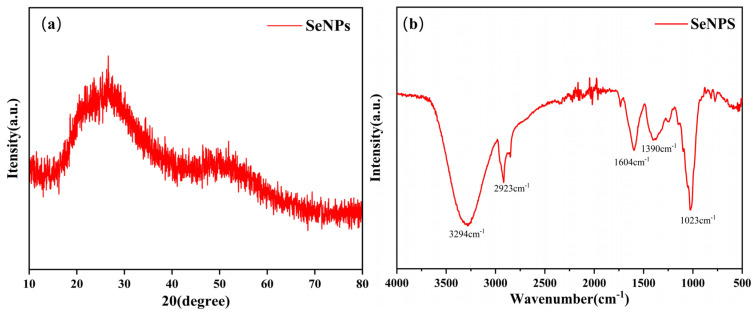
Structural and functional characterization of green-synthesized selenium nanoparticles (SeNPs). (**a**) X-ray diffraction (XRD) pattern, and (**b**) Fourier-transform infrared (FTIR) spectrum.

The crystalline structure of the synthesized SeNPs was examined using X-ray diffraction (XRD). The XRD pattern exhibited broad diffraction features with a dominant peak at 2θ = 26.5°, corresponding to the (101) lattice plane of elemental selenium (JCPDS card no. 06-0362), confirming the formation of selenium nanoparticles (Figure 3a). The broadness and relatively low intensity of the diffraction peaks indicate that the nanoparticles predominantly possess an amorphous or poorly crystalline structure. Amorphous SeNPs are commonly produced via green synthesis approaches because the rapid reduction of selenium ions by plant-derived biomolecules limits the ordered crystal growth process [11]. Such structural characteristics have been widely reported for biogenic SeNPs synthesized using plant extracts and microbial systems.

The amorphous nature of SeNPs is particularly advantageous for biological and environmental applications. Compared with crystalline selenium, amorphous SeNPs generally exhibit higher surface reactivity, improved bioavailability, and enhanced antioxidant capacity due to their higher surface energy and greater density of reactive surface sites [12]. These properties can significantly influence nanoparticle interactions with plant roots and cellular systems. In plant stress physiology, nanoscale selenium has been reported to enhance antioxidant defense mechanisms, regulate reactive oxygen species (ROS) metabolism, and reduce oxidative damage caused by heavy metal stress [38]. Therefore, the amorphous structure of the synthesized SeNPs may contribute to their higher biological activity and effectiveness in mitigating Cr toxicity.

To further investigate the surface chemistry of the SeNPs, Fourier-transform infrared spectroscopy (FTIR) analysis was conducted (Figure 3b). The FTIR spectrum displayed a broad absorption band around 3294 cm^−1^, which is attributed to O–H and N–H stretching vibrations, indicating the presence of hydroxyl and amine groups derived from plant biomolecules. A distinct peak at 2923 cm^−1^ corresponds to C–H stretching vibrations associated with aliphatic hydrocarbons. The strong absorption band at 1604 cm^−1^ is assigned to C=O stretching vibrations (amide I), suggesting the presence of proteins or peptide residues that may act as stabilizing ligands. Additional peaks observed at 1390 cm^−1^ and 1023 cm^−1^ correspond to C–H bending and C–O/Se–O stretching vibrations, respectively, which are commonly associated with polysaccharides, phenolic compounds, and other oxygen-containing functional groups [9,17].

These functional groups originate from the phytochemical components of banana peel extract, including polyphenols, carbohydrates, proteins, and organic acids, which play crucial roles during nanoparticle synthesis. During the green synthesis process, these biomolecules act simultaneously as reducing agents that convert selenium ions into elemental selenium and as capping agents that stabilize the nanoparticles by forming a protective organic corona on their surface [32]. This organic corona enhances nanoparticle stability by preventing aggregation through steric hindrance and electrostatic interactions.

More importantly, the presence of oxygen-containing functional groups on the nanoparticle surface may provide active binding sites for heavy metal ions. Functional groups such as hydroxyl, carboxyl, and carbonyl moieties can interact with metal ions through complexation, electrostatic attraction, or surface adsorption mechanisms [43]. These interactions can facilitate the immobilization or reduction of toxic metal species, thereby contributing to detoxification processes. In the context of chromium stress, the organic coating surrounding SeNPs may assist in binding or reducing Cr species while simultaneously enhancing plant antioxidant responses, representing a potential mechanism for alleviating heavy metal toxicity [44].

### 3.2. Physiological and Biochemical Effects of SeNPs on I. aquatica Under Chromium Stress

#### 3.2.1. SeNPs Mitigate cr-Induced Growth Inhibition and Biomass Reduction

**Figure 4:**
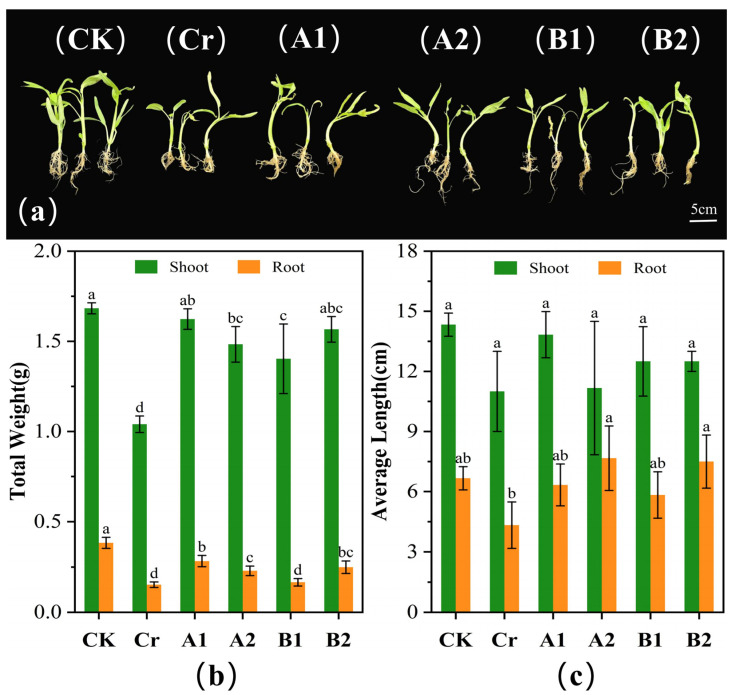
Effects of selenium treatments on the growth performance of *I. aquatica* under Cr(VI) stress. (**a**) Representative images of plants, (**b**) total biomass (shoot and root) and (**c**) average length of shoots and roots under different treatments. Treatments: control (CK), Cr(VI) stress (Cr) (20 mg L^−1^), SeNPs at 10 mg L^−1^ (A1) and 1 mg L^−1^ (A2), and Na_2_SeO_3_ at 10 mg L^−1^ (B1) and 1 mg L^−1^ (B2). Data are presented as mean ± standard deviation (n = 3). Different letters indicate significant differences among treatments (*p* < 0.05).

Chromium exposure significantly inhibited the growth of *I. aquatica*. Compared with the control (CK), plants subjected to Cr stress (Cr group) exhibited marked reductions in shoot length (23.0%), root length (35.8%), shoot biomass (38.0%), and root biomass (60.0%) (Figure 4a–c). These results indicate severe phytotoxic effects of chromium on plant development. Chromium toxicity has been widely reported to impair root cell division, disrupt membrane integrity, and interfere with nutrient and water uptake, ultimately leading to reduced plant growth and biomass accumulation [45,46]. In particular, hexavalent chromium (Cr(VI)) can enter plant cells via sulfate or phosphate transporters, where it is subsequently reduced to Cr(III), generating excessive reactive oxygen species (ROS) and inducing oxidative stress that damages cellular components including proteins, lipids, and nucleic acids [38,46].

The application of SeNPs significantly alleviated the inhibitory effects of chromium stress (Figure 4a–c). Treatment with 10 mg L^−1^ SeNPs (A1) almost completely restored shoot and root growth, with values approaching those of the control plants. This finding indicates that SeNPs effectively mitigated chromium-induced phytotoxicity. In contrast, the application of sodium selenite (10 mg L^−1^, B1) produced a comparatively weaker recovery effect, suggesting that nano-selenium exhibits higher physiological efficacy than its ionic counterpart. The enhanced performance of SeNPs is attributed to their nanoscale characteristics such as a higher surface-to-volume ratio, increased surface area, unique surface reactivity, improved bioavailability, and gradual selenium release which collectively enhance their interaction with plant tissues, resulting in greater biological activity and antioxidative capacity [47,48].

Interestingly, a low concentration of SeNPs (1 mg L^−1^, A2) not only alleviated chromium toxicity but also promoted root growth, resulting in a 14.9% increase in root length compared with the chromium-supplemented control. This observation suggests that low-dose SeNPs function as plant growth stimulants under stress conditions, as selenium at low concentrations enhances antioxidant capacity, regulates phytohormone balance, promotes root development, and upregulates antioxidant enzymes (SOD, CAT, APX, POD, GPX, and GR), thereby reducing MDA, and other oxidative stress markers in plants [49]. The stimulatory effect observed in this study may therefore be associated with improved cellular redox homeostasis and enhanced metabolic activity.

#### 3.2.2. SeNPs Protect Photosynthetic Pigments from Cr-Induced Degradation

Chromium exposure caused a pronounced decline in photosynthetic pigments in *I. aquatica*. Compared with the control, the Cr treatment reduced chlorophyll a by 22.6%, chlorophyll b by 32.3%, and total chlorophyll by 27.9% (Figure S3), indicating substantial impairment of the photosynthetic machinery. Such reductions in chlorophyll content are a common physiological response to heavy metal stress and reflect damage to chloroplast structure and pigment biosynthesis pathways. Chromium has been reported to disrupt chloroplast ultrastructure, inhibit chlorophyll biosynthesis by interfering with magnesium incorporation into the porphyrin ring, and promote pigment degradation through oxidative damage [50]. In addition, Cr-induced reactive oxygen species (ROS) can oxidize membrane lipids and destabilize thylakoid membranes, leading to the breakdown of chlorophyl-protein complexes and decreased photosynthetic efficiency [38].

The application of selenium, particularly in nanoparticle form, significantly alleviated this decline in chlorophyll content. Both SeNPs and sodium selenite treatments improved pigment levels compared with the Cr-only treatment, but the effect was more pronounced for SeNPs. The treatment with 10 mg L^−1^ SeNPs (A1) restored total chlorophyll content to 94.8% of the control level, suggesting a strong protective role of nano-selenium in preserving the photosynthetic apparatus under chromium stress. This improvement may be attributed to several physiological and biochemical mechanisms. Selenium has been shown to enhance the antioxidant defense system in plants by stimulating the activity of enzymes such as SOD, CAT, POD, which collectively reduce ROS accumulation and protect chloroplast structures from oxidative damage [38]. Another possible mechanism is that selenium stabilizes chloroplast membranes and protects the photosynthetic electron transport chain by limiting lipid peroxidation and preserving PSII pigment-protein complexes, thereby maintaining chloroplast ultrastructure and thylakoid organization under stress conditions (salt, drought, temperature, and metal), which ultimately supports higher chlorophyll content and photosynthetic efficiency [51]. In addition, selenium may regulate the expression of genes involved in chlorophyll biosynthesis and photosynthetic metabolism, thereby supporting pigment regeneration and maintaining photosynthetic capacity under heavy metal stress, as evidenced by the upregulation of key porphyrin/chlorophyll biosynthesis genes under Cd stress in rice and the restoration of chloroplast development-related gene expression under Pb stress in pakchoi, which collectively enhance chlorophyll and carotenoid synthesis and recover photosynthetic performance [13,52].

#### 3.2.3. SeNPs Restore Mineral Nutrient Homeostasis Disrupted by Cr

Chromium exposure significantly disrupted the uptake and accumulation of essential macronutrients in *I. aquatica*, leading to marked reductions in nitrogen (N), phosphorus (P), potassium (K), calcium (Ca), and magnesium (Mg) contents in plant tissues (Figure S4). Such nutrient imbalances are commonly reported under heavy metal stress and reflect impaired root function and transporter activity. Chromium ions disrupt nutrient uptake by competing with essential ions for transport pathways and binding sites on root membranes while impairing membrane integrity and ion channel function, with Cr(VI) specifically entering roots via phosphate and sulfate transporters and directly competing with phosphate (P) and sulfate (S) for carrier binding [50]. In particular, Cr(VI) may enter plant roots through sulfate or phosphate transporters due to its structural similarity to these anions, thereby interfering with the normal uptake of phosphorus and other nutrients [53]. Moreover, chromium-induced oxidative stress can disrupt plasma membrane permeability and inhibit the activity of key transport proteins responsible for nutrient absorption and translocation [38]. As a result, plants exposed to chromium often experience deficiencies in essential mineral elements, which further aggravates growth inhibition and metabolic dysfunction.

The application of SeNPs markedly alleviated these nutrient imbalances. Treatment with 10 mg L^−1^ SeNPs (A1) restored the concentrations of N, P, K, Ca, and Mg to levels close to those observed in the control plants, demonstrating the strong capacity of SeNPs to maintain mineral nutrient homeostasis under chromium stress. In contrast, the sodium selenite treatment showed a comparatively weaker recovery effect, indicating that nano-selenium possesses greater physiological efficacy than its ionic form. This enhanced performance is attributed to the unique physicochemical properties of SeNPs such as high surface reactivity, improved bioavailability, small size, and sustained selenium release which enable efficient uptake, enhanced antioxidant activity, and prolonged protective effects in plants compared to conventional selenium forms [14,48].

#### 3.2.4. The Impact of SeNPs on Antioxidant Enzymes

**Figure 5:**
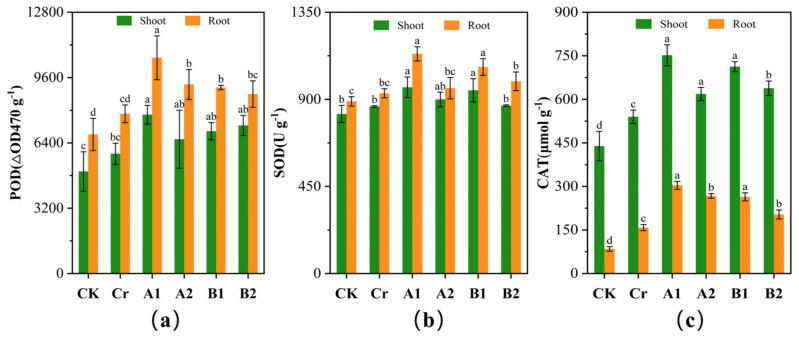
Effects of selenium treatments on antioxidant enzyme activities in *I. aquatica* under Cr(VI) stress. (**a**) Peroxidase (POD), (**b**) superoxide dismutase (SOD), and (**c**) catalase (CAT) activities in shoots and roots under different treatments. Different letters indicate significant differences among treatments (*p* < 0.05).

Chromium exposure triggered a pronounced oxidative stress response in *I. aquatica*, as evidenced by the increased activities of key antioxidant enzymes, including SOD, PODandCAT (Figure 5a–c). The activation of these enzymes represents a typical plant defense mechanism against excessive reactive oxygen species generated under heavy metal stress. Cr(VI) stress induces excessive ROS (OH) that damage cellular components, and plants counter this by activating antioxidant defenses (e.g., SOD, CAT, peroxidases) along with non-enzymatic antioxidants and metal-chelating compounds to detoxify ROS and maintain redox balance [54,55]. In response, plants enhance the activity of antioxidant enzymes to detoxify ROS and maintain cellular redox balance.

The application of selenium significantly amplified this antioxidant defense response. Both selenium treatments enhanced enzyme activities compared with the Cr-only treatment; however, SeNPs exhibited a substantially stronger effect than sodium selenite. In the roots of plants treated with 10 mg L^−1^ SeNPs (A1), the activities of SOD, POD, and CAT increased by 24.0%, 35.0%, and 91.9%, respectively, relative to the Cr-stressed plants. The increase in SOD activity indicates enhanced dismutation of superoxide radicals into hydrogen peroxide, while elevated CAT and POD activities facilitate the rapid conversion of hydrogen peroxide into water and oxygen, thereby preventing the accumulation of toxic ROS [38]. Such coordinated activation of antioxidant enzymes suggests that SeNPs play a crucial role in reinforcing the plant antioxidant network and mitigating oxidative damage caused by chromium stress. Selenium is widely recognized for its ability to modulate plant redox metabolism and enhance antioxidant capacity. One key mechanism involves selenium incorporation into selenoproteins such as glutathione peroxidase (GPX), which catalyzes the reduction of hydrogen peroxide and lipid hydroperoxides to water or alcohols using glutathione as an electron donor, thereby maintaining cellular redox balance and protecting against oxidative damage [56,57]. This reaction forms an essential component of the ascorbate–glutathione cycle, a major cellular pathway responsible for maintaining ROS homeostasis in plant cells. By enhancing the activity of GPX and related antioxidant enzymes, selenium improves the detoxification of ROS such as hydrogen peroxide and lipid hydroperoxides, thereby maintaining redox balance and protecting cellular membranes from oxidative damage [58,59].

The superior performance of SeNPs compared with sodium selenite may be attributed to their nanoscale properties and improved physiological availability. Nanoparticles generally exhibit higher surface area and reactivity, allowing them to interact more efficiently with plant tissues and cellular components. Additionally, SeNPs exhibit sustained-release behavior, gradually supplying bioavailable selenium to plant cells, which supports prolonged activation of antioxidant pathways, maintains higher antioxidant enzyme activities (e.g., SOD, CAT, GPX), reduces ROS and lipid peroxidation, and improves stress tolerance and physiological performance more effectively than conventional selenium forms [14,15]. The organic biomolecules that coat green-synthesized SeNPs may also enhance nanoparticle stability and facilitate their transport within plant tissues, further contributing to their enhanced biological effectiveness [9].

#### 3.2.5. SeNPs Alleviate Oxidative Damage and Osmotic Stress

**Figure 6:**
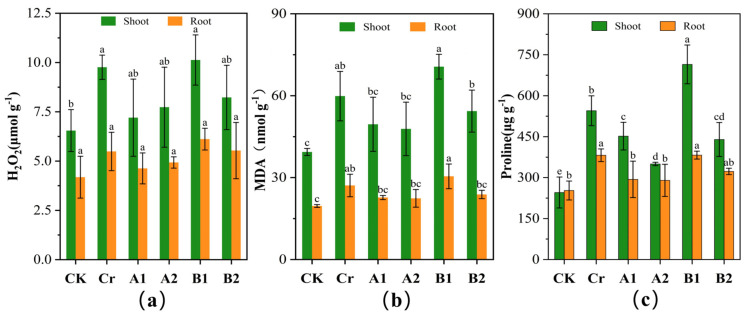
Effects of selenium treatments on oxidative stress indicators and osmolyte accumulation in *I. aquatica* under Cr(VI) stress. (**a**) Hydrogen peroxide (H_2_O_2_) content, (**b**) malondialdehyde (MDA) content, and (**c**) proline content in shoots and roots under different treatments. Different letters indicate significant differences among treatments (*p* < 0.05).

Chromium stress caused pronounced oxidative damage in *I. aquatica*, as indicated by the substantial accumulation of hydrogen peroxide and MDA (Figure 6a–c). Hydrogen peroxide is a major ROS produced during heavy metal stress, while MDA is a well-established biomarker of lipid peroxidation and membrane damage [38,60]. The elevated levels of these compounds in the Cr-treated plants suggest severe disruption of cellular redox homeostasis and structural damage to membrane lipids. Chromium-induced ROS accumulation can trigger peroxidation of polyunsaturated fatty acids in cellular membranes, leading to increased membrane permeability, loss of ion balance, and impairment of metabolic processes [61]. Such oxidative damage ultimately compromises cell integrity and physiological functioning.

In addition to oxidative stress, chromium exposure also induced osmotic imbalance, as reflected by the sharp increase in proline content observed in both leaves and roots. Proline is a key that accumulates under abiotic stress (e.g., drought, salinity, heavy metals), where it stabilizes proteins and membranes, maintains osmotic balance, scavenges ROS, and enhances antioxidant defense and metabolic regulation, thereby contributing to improved stress tolerance in plants [62,63]. The increased proline levels in the Cr treatment therefore represent an adaptive response aimed at mitigating osmotic and oxidative stress. However, excessive accumulation of proline is often associated with severe stress conditions and metabolic disturbances in plants exposed to heavy metals.

The application of SeNPs significantly alleviated these stress responses. Treatment with 10 mg L^−1^ SeNPs (A1) markedly reduced the concentrations of MDA, and proline in both leaves and roots compared with the Cr-only treatment, indicating effective mitigation of oxidative damage and restoration of cellular homeostasis. These findings are consistent with the well-documented antioxidant role of selenium in plants. Selenium enhances the activity of antioxidant enzymes (e.g., CAT, GPx, APX, GR), thereby reducing ROS accumulation, limiting lipid peroxidation, and protecting cellular membranes from oxidative damage through improved redox regulation [64,65]. The reduction in proline content observed in SeNP-treated plants further suggests that the severity of stress was alleviated, reducing the need for osmotic adjustment mechanisms. In contrast, treatment with a high concentration of sodium selenite (10 mg L^−1^, B1) aggravated oxidative damage in leaves, resulting in higher levels of MDA compared with the SeNP treatment. This observation highlights the narrow beneficial concentration range of ionic selenium forms and their potential phytotoxicity at elevated concentrations. Ionic selenium species such as selenite can rapidly accumulate in plant tissues and interfere with sulfur metabolism, leading to the formation of toxic intermediate compounds and additional oxidative stress [65]. Consequently, excessive selenite may induce secondary toxicity rather than providing protective effects.

#### 3.2.6. SeNPs Effect on Primary Metabolic Processes

**Figure 7:**
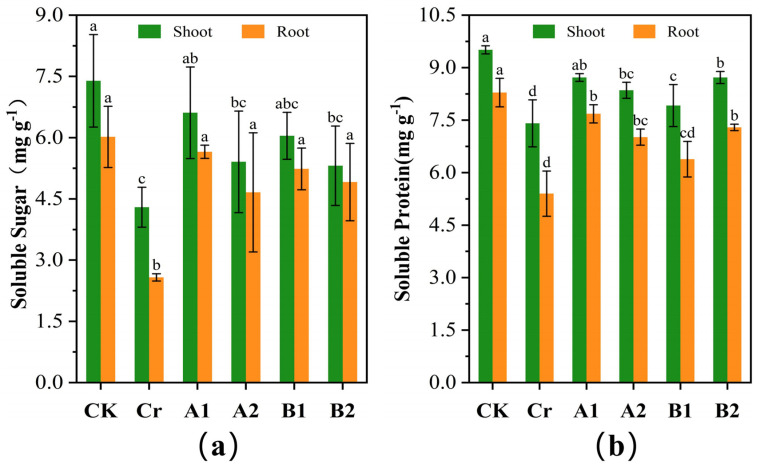
Effects of selenium treatments on primary metabolite accumulation in *I. aquatica* under Cr(VI) stress. (**a**) Soluble sugar and (**b**) soluble protein contents in shoots and roots under different treatments. Different letters indicate significant differences among treatments (*p* < 0.05).

Chromium stress significantly disrupted primary metabolic processes in *I. aquatica*, as reflected by the marked reductions in soluble protein and soluble sugar contents (Figure 7). These metabolites play essential roles in plant growth and energy metabolism, and their decline indicates severe physiological impairment under heavy metal stress. Chromium toxicity disrupts key metabolic pathways by impairing photosynthesis, inhibiting enzyme activities involved in nitrogen and carbohydrate metabolism, and inducing ROS-mediated oxidative damage, ultimately reducing chlorophyll synthesis, protein formation, and carbohydrate production, and thereby limiting plant growth and productivity [46,66]. Cr toxicity damages chloroplasts and disrupts the photosynthetic electron transport chain, reducing carbon fixation and carbohydrate production, while ROS-induced oxidative stress and nutrient imbalance impair amino acid metabolism and protein biosynthesis, collectively limiting photosynthetic efficiency and metabolic function [67]. Consequently, decreased levels of soluble sugars and proteins are commonly observed in plants exposed to chromium contamination and reflect the suppression of both carbon and nitrogen metabolism.

The application of SeNPs significantly alleviated these metabolic disruptions. Treatment with 10 mg L^−1^ SeNPs (A1) effectively restored soluble protein and soluble sugar contents to 91.5% and 89.1% of control levels in shoots, respectively, outperforming all other selenium treatments. This result indicates that nano-selenium plays an important role in stabilizing metabolic pathways under chromium stress. The restoration of soluble sugars suggests an improvement in photosynthetic efficiency and carbohydrate metabolism, while the recovery of protein levels reflects enhanced nitrogen assimilation and enzyme synthesis. Selenium enhances the activity of enzymes involved in carbon fixation and nitrogen metabolism (e.g., nitrate reductase, glutamine synthetase), thereby improving photosynthesis, amino acid and carbohydrate synthesis, and overall metabolic performance under stress conditions [68,69].

Several mechanisms may explain the beneficial effects of SeNPs on primary metabolism. First, selenium can mitigate oxidative damage by enhancing antioxidant defenses, thereby protecting metabolic enzymes and cellular organelles from ROS-induced degradation [38]. Second, by preserving chloroplast integrity and photosynthetic activity, selenium indirectly supports carbohydrate production and energy metabolism [61]. Third, the improved nutrient uptake observed in SeNP-treated plants may contribute to enhanced nitrogen and phosphorus availability, which are essential for protein synthesis and metabolic regulation. The nanoscale properties of SeNPs enhance selenium bioavailability, stability, and controlled release within plant tissues, enabling more efficient metabolic regulation, antioxidant activity, and enzyme function compared with ionic selenium forms while reducing toxicity risks [70,71]. Overall, the recovery of soluble sugars and proteins indicates that SeNPs effectively restore primary metabolic processes disrupted by chromium stress. This systemic recovery of carbon and nitrogen metabolism reflects improved physiological functioning and overall plant health. The results demonstrate that the protective effects of SeNPs extend beyond antioxidant defense to include the stabilization of fundamental metabolic pathways, highlighting their potential role in enhancing plant resilience in heavy metal-contaminated environments.

#### 3.2.7. SeNPs Effect on Cr Accumulation and Se Biofortification

**Figure 8:**
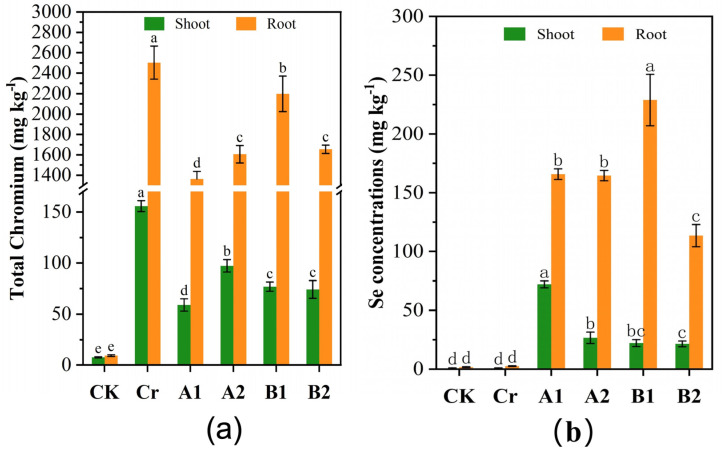
Effects of selenium treatments on chromium (Cr) accumulation and selenium (Se) biofortification in *I. aquatica* under Cr(VI) stress. (**a**) Total Cr concentrations and (**b**) Se concentrations in shoots and roots under different treatments. Different letters indicate significant differences among treatments (*p* < 0.05).

A key outcome of this study was the regulation of Cr and Se accumulation and translocation in *I. aquatica* (Figure 8a,b). Under Cr stress, plants predominantly accumulate Cr in root tissues, which act as the primary barrier limiting its translocation to aerial parts through sequestration in cell walls and vacuoles, activation of antioxidant defenses, and regulation of stress-related genes, thereby reducing toxicity in shoots and contributing to overall tolerance [72,73]. Root retention of Cr is generally associated with adsorption to cell wall components such as pectin and lignin, as well as sequestration within root vacuoles, which collectively restrict metal transport to shoots [60]. However, despite this natural defense mechanism, substantial Cr translocation to edible tissues can still occur under high exposure levels, posing potential food safety risks.

The application of SeNPs significantly reduced Cr accumulation in both roots and shoots compared with the Cr-only treatment (Figure 8a). Notably, treatment with 10 mg L^−1^ SeNPs (A1) decreased Cr concentration in the edible shoots by 45.4%, demonstrating a strong inhibitory effect on root-to-shoot Cr translocation. This reduction was substantially greater than that observed in the sodium selenite treatment, indicating the superior capacity of nano-selenium to regulate heavy metal uptake and transport. Several mechanisms may explain this effect. First, the functional groups present on the surface of SeNPs originating from biomolecules in the banana peel extract may adsorb or chelate Cr ions in the rhizosphere, thereby reducing their bioavailability and uptake by plant roots [28]. Second, selenium facilitates the formation of insoluble or less mobile Cr Se complexes within root tissues, which are subsequently sequestered in vacuoles or bound to cell wall components, thereby limiting Cr translocation to shoots; this process is further supported by improved root structural integrity and regulation of metal transport and stress-related genes [74,75]. Third, selenium has been reported to regulate the expression of genes associated with heavy metal transport and detoxification, potentially downregulating transporters responsible for Cr uptake and long-distance transport [61,76]. In addition to reducing chromium accumulation, SeNPs significantly enhanced selenium enrichment in plant tissues. Plants treated with SeNPs accumulated substantially higher Se concentrations in the shoots compared with those treated with sodium selenite, which tended to retain selenium primarily in the roots. This differential distribution pattern suggests that nano-selenium exhibits improved mobility and bioavailability within plant tissues. The nanoscale size and controlled-release properties of SeNPs facilitate their uptake and vascular transport, allowing gradual conversion into bioavailable forms and more efficient delivery of selenium to aerial tissues compared with ionic forms, thereby enhancing accumulation and biofortification [77,78]. Moreover, the organic coating derived from plant extracts may improve nanoparticle stability and interaction with plant membranes, further promoting selenium transport and assimilation.

### 3.3. Statistical Correlation of Physiological, Biochemical, and Morphological Parameters

**Figure 9:**
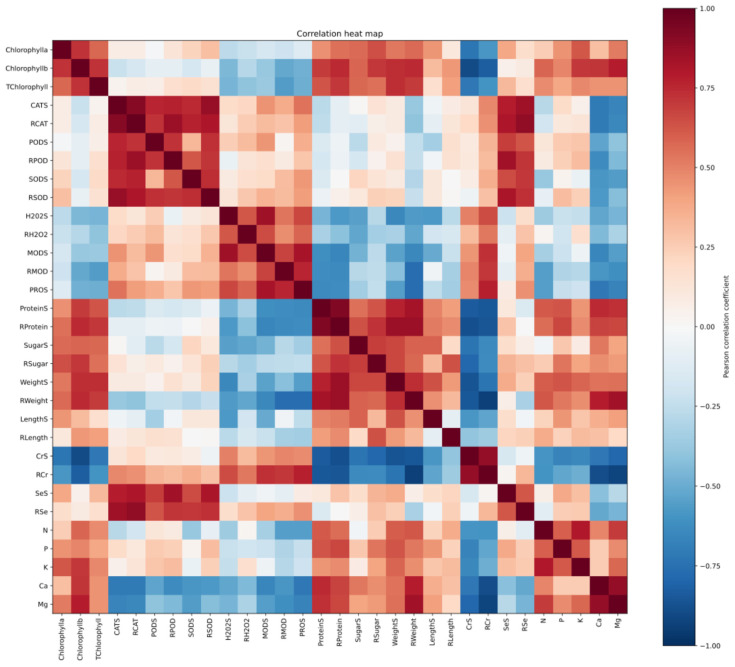
Pearson correlation heatmap illustrating relationships among physiological, biochemical, growth, and elemental parameters of *I. aquatica* under different treatments. Color intensity represents correlation coefficients (red: positive, blue: negative).

The correlation matrix presented in Figure 9 reveals a strong association among the physiological, biochemical, and growth parameters of *I. aquatica* subjected to chromium stress and various selenium source treatments. Notably, chromium content exhibits a significant negative correlation with growth, photosynthesis, and metabolic indicators, including plant length, weight, chlorophyll content, soluble sugars, protein, and nutrient element content. This finding substantiates that Cr(VI) impedes the growth of *I. aquatica* by inhibiting photosynthesis and metabolic processes. Conversely, chromium content shows a positive correlation with oxidative stress markers, such as H_2_O_2_ and MOD, as well as with antioxidant enzyme activities, including CAT and POD. This relationship confirms that chromium induces oxidative damage and activates the plant’s antioxidant defense mechanisms related to oxidase activity.

The selenium content in the aboveground parts (SeS) exhibits a strong positive correlation with the activities of antioxidant enzymes (CAT, POD), proteins, and soluble sugars, while it shows a negative correlation with CrS. This finding indicates that SeNPs effectively inhibit the transport of chromium to the aboveground parts and mitigate its toxicity by promoting the antioxidant system and enhancing plant metabolic activities. Furthermore, the levels of soluble sugars, proteins, and nutrient elements are positively correlated with chlorophyll content, suggesting a protective effect on photosynthesis during the chromium stress response. Collectively, these correlations elucidate the toxic mechanism of chromium on *I. aquatica* and confirm that exogenous selenium can enhance the antioxidant defense capacity of plants, reduce oxidative damage, and alleviate the toxic effects of chromium. This provides a robust theoretical foundation for the application of nano-selenium as an in hydroponic systems contaminated by heavy metals.

### 3.4. Possible Mechanism of SeNPs-Mediated Chromium Detoxification in I. aquatica

**Figure 10:**
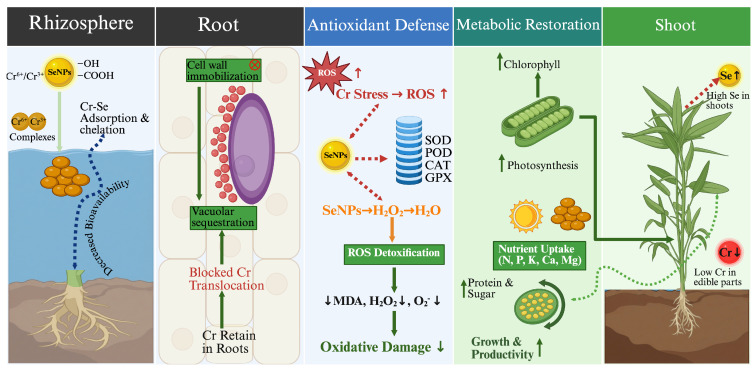
Proposed mechanism of selenium nanoparticle (SeNP)-mediated chromium detoxification in *I. aquatica*.

Based on the physiological, biochemical, and elemental analyses obtained in this study, a conceptual mechanism is proposed to explain the protective effects of selenium nanoparticles (SeNPs) against chromium (Cr) toxicity in *I. aquatica* (Figure 10). It should be noted that the mechanisms described below are inferred from the observed physiological responses together with evidence from previous studies and should therefore be regarded as proposed mechanisms rather than direct experimental evidence.

First, SeNPs may reduce the bioavailability of Cr in the rhizosphere. Green-synthesized SeNPs contain surface functional groups, including hydroxyl, carboxyl, and carbonyl groups derived from plant biomolecules, which have been reported to adsorb or interact with heavy metal ions, thereby potentially reducing the concentration of bioavailable Cr species surrounding the roots [28]. Previous studies have also suggested that selenium may promote the formation of less mobile Cr-containing complexes or facilitate Cr immobilization within root tissues, thereby reducing its mobility and toxicity [79,80]. Although these processes were not directly examined in the present study, the significantly lower Cr accumulation observed in SeNP-treated plants is consistent with these proposed mechanisms.

Second, SeNPs appeared to restrict Cr translocation from roots to shoots. Most Cr accumulated in the roots, while SeNP application significantly reduced its accumulation in edible shoots. This finding suggests that SeNPs may enhance Cr retention within root tissues through cell wall binding and/or vacuolar compartmentalization, as proposed in previous studies [76]. However, because cellular localization and transporter gene expression were not investigated, these processes should be regarded as plausible hypotheses requiring further validation.

Third, SeNP treatment markedly enhanced the activities of SOD, POD, and CAT while reducing H_2_O_2_ and MDA accumulation, indicating that improved antioxidant capacity is an important component of Cr tolerance. Previous studies have suggested that selenium can participate in cellular redox regulation through multiple antioxidant pathways, including selenoprotein-mediated antioxidant systems and the ascorbate–glutathione cycle [38]. However, these pathways were not directly investigated in the present study, and therefore their involvement is inferred from the observed physiological responses and published literature rather than experimentally demonstrated.

Finally, SeNP application restored chlorophyll content, nutrient uptake, and primary metabolism under Cr stress. These improvements indicate that SeNPs effectively alleviated Cr-induced physiological dysfunction and helped maintain photosynthetic performance and nutrient homeostasis. Although previous studies have associated these responses with the regulation of chlorophyll biosynthesis, nutrient transport, and metabolic pathways [61], the present study did not examine gene expression or transporter activity. Consequently, these molecular mechanisms remain hypothetical and require confirmation through transcriptomic, proteomic, and biochemical analyses. Overall, the present results demonstrate that green-synthesized SeNPs effectively alleviated Cr toxicity by reducing Cr accumulation, enhancing antioxidant capacity, maintaining nutrient homeostasis, and restoring plant growth and metabolism. While the proposed mechanisms are supported by the previous literature and are consistent with our experimental observations, further studies integrating metal speciation, gene expression, antioxidant pathway analysis, and transporter characterization are needed to verify the molecular and biochemical mechanisms underlying SeNP-mediated Cr detoxification.

## 4. Conclusions

Developing sustainable strategies to reduce heavy metal accumulation in food crops while maintaining crop productivity is essential for ensuring agricultural sustainability and food safety. In this study, green-synthesized selenium nanoparticles (SeNPs) derived from banana peel extract and conventional sodium selenite both effectively alleviated chromium (Cr)-induced phytotoxicity in *I. aquatica* grown under a dynamic cyclic hydroponic system. Both selenium forms improved plant growth, photosynthetic performance, antioxidant defense, nutrient homeostasis, and primary metabolism compared with Cr treatment alone. Although SeNPs generally produced greater improvements across most measured parameters, statistically significant differences between SeNPs and sodium selenite were primarily observed for chromium accumulation, where SeNPs more effectively reduced Cr translocation to edible shoots while simultaneously enhancing selenium biofortification. These findings suggest that green-synthesized SeNPs represent a promising and sustainable selenium source for improving crop safety and nutritional quality under chromium stress.

The use of banana peel as a renewable precursor further supports agricultural waste valorization and circular economy principles. However, the present study was conducted under controlled hydroponic conditions and focused on short-term plant responses. Therefore, further research is required to evaluate the long-term stability, environmental fate, and full life-cycle effects of SeNPs, as well as their performance under soil-based and field cultivation systems. Comparative studies in static and dynamic hydroponic systems and investigations of nanoparticle behavior, metal speciation, and rhizosphere processes will further improve our understanding of the mechanisms underlying SeNP-mediated alleviation of Cr(VI) toxicity and support their safe and practical application in sustainable agriculture.

## Data Availability

The original contributions presented in this study are included in the article/Supplementary Material. Further inquiries can be directed to the corresponding authors.

## Supplementary Materials

The following supporting information can be downloaded at: https://www.mdpi.com/article/10.3390/plants15152279/s1, Figure S1: Physicochemical characterization of green-synthesized selenium nanoparticles (SeNPs). (a) Dynamic light scattering (DLS) size distribution, and (b) Zeta potential; Figure S2: Elemental composition and spatial distribution of green-synthesized selenium nanoparticles (SeNPs) analyzed by energy-dispersive X-ray spectroscopy (EDS); Figure S3: Effects of selenium treatments on chlorophyll content of water spinach under Cr(VI) stress. Chlorophyll a, chlorophyll b, and total chlorophyll contents were measured under different treatments. Treatments: control (CK), Cr(VI) stress (Cr) (20 mg L^−1^), SeNPs at 1 mg L^−1^ (A1) and 10 mg L^−1^ (A2), and Na_2_SeO_3_ at 1 mg L^−1^ (B1) and 10 mg L^−1^ (B2). Data are presented as mean ± standard deviation (n = 3). Different letters indicate significant differences among treatments (*p* < 0.05).; Figure S4: Effects of selenium treatments on macro- and micronutrient contents (N, P, K, Ca, and Mg) in water spinach under Cr(VI) stress.

## List of Abbreviations

**Abbreviation**	**Full Term**
ABA	Abscisic Acid
ANOVA	Analysis of Variance
AsA	Ascorbic Acid
AsA-GSH	Ascorbate–Glutathione
CAT	Catalase
CK	Control
Cr	Chromium
Cr(VI)	Hexavalent Chromium
DLS	Dynamic Light Scattering
DW	Dry Weight
GPX	Glutathione Peroxidase
H_2_O_2_	Hydrogen Peroxide
ICP-MS	Inductively Coupled Plasma Mass Spectrometry
MDA	Malondialdehyde
Na_2_SeO_3_	Sodium Selenite
NPs	Nanoparticles
POD	Peroxidase
ROS	Reactive Oxygen Species
SD	Standard Deviation
Se	Selenium
SeNPs	Selenium Nanoparticles
SOD	Superoxide Dismutase
TEM	Transmission Electron Microscopy
XRD	X-ray Diffraction
K_2_Cr_2_O_7_	Potassium dichromate

## References

[1] Hussein S.H., Qurbani K., Ahmed S.K., Tawfeeq W., Hassan M. (2024). Bioremediation of heavy metals in contaminated environments using Comamonas species: A narrative review. Bioresour. Technol. Rep..

[2] Kanwar V.S., Sharma A., Srivastav A.L., Rani L. (2020). Phytoremediation of toxic metals present in soil and water environment: A critical review. Environ. Sci. Pollut. Res..

[3] Ijaz S., Iqbal J., Abbasi B.A., Tufail A., Ullah Z., Yaseen T., Ali I., Uddin S., Iqbal R. (2024). Heavy metal–polluted arable land and its consequences: A global scenario. Biochar-Assisted Remediation of Contaminated Soils Under Changing Climate.

[4] Abd Elnabi M.K., Elkaliny N.E., Elyazied M.M., Azab S.H., Elkhalifa S.A., Elmasry S., Mouhamed M.S., Shalamesh E.M., Alhorieny N.A., Abd Elaty A.E. (2023). Toxicity of heavy metals and recent advances in their removal: A review. Toxics.

[5] Milla O.V., Rivera E.B., Huang W. (2014). Bioaccumulations of heavy metals in *Ipomoea aquatica* grown in bottom ash recycling wastewater. Water Environ. Res..

[6] Haokip N., Gupta A. (2021). Biochemical and antioxidant responses of *Ipomoea aquatica* exposed to graded concentrations of chromium. Biologia.

[7] Sahoo R., Sow S., Ranjan S., Dharminder, Kumar R., Roy D.K., Kumar S., Kumar A., Srivastava R.K., Prasad R. (2024). Unveiling the potential of plant growth promoting rhizobacteria (PGPR) in phytoremediation of heavy metal. Discov. Appl. Sci..

[8] Tang T., Liu X., Wang L., Zuh A.A., Qiao W., Huang J. (2020). Uptake, translocation and toxicity of chlorinated polyfluoroalkyl ether potassium sulfonate (F53B) and chromium co-contamination in water spinach (*Ipomoea aquatica Forsk*). Environ. Pollut..

[9] Iravani S. (2011). Green synthesis of metal nanoparticles using plants. Green Chem..

[10] Bhattacharjee S. (2016). DLS and zeta potential—What they are and what they are not?. J. Control. Release.

[11] Li K., Li J., Zhang S., Zhang J., Xu Q., Xu Z., Guo Y. (2024). Amorphous structure and crystal stability determine the bioavailability of selenium nanoparticles. J. Hazard. Mater..

[12] Geng L., Li L., Sun X., Cheng S., He J. (2025). Recent advances towards selenium nanoparticles: Synthetic methods, functional mechanisms, and biological applications. Foods.

[13] Tan Z., Xuan Z., Wu C., Cheng Y., Xu C., Ma X., Wang D. (2022). Effects of selenium on the AsA-GSH system and photosynthesis of pakchoi (*Brassica chinensis* L.) under lead stress. J. Soil Sci. Plant Nutr..

[14] El-Badri A.M., Hashem A.M., Batool M., Sherif A., Nishawy E., Ayaad M., Hassan H.M., Elrewainy I.M., Wang J., Kuai J. (2022). Comparative efficacy of bio-selenium nanoparticles and sodium selenite on morpho-physiochemical attributes under normal and salt stress conditions, besides selenium detoxification pathways in *Brassica napus* L.. J. Nanobiotechnol..

[15] Abbas S., Nidhi, Anjum N.A., Fatma M., Ahmad A. (2025). Assessment of the major biochemical and physiological mechanisms underlying green-synthesised selenium nanoparticles-mediated alleviation of lanthanum toxicity in mung bean (*Vigna radiata* L.). J. Hazard. Mater..

[16] Wang X., Wu G., Wang Y., Lu M., Guo Y., Yin W., Sun C., Chen Y., Yin X. (2024). Selenium enhancement strategy under precise fertilization in foxtail millet rhizosphere. Heliyon.

[17] Sagar N.A., Pareek S., Sharma S., Yahia E.M., Lobo M.G. (2018). Fruit and vegetable waste: Bioactive compounds, their extraction, and possible utilization. Compr. Rev. Food Sci. Food Saf..

[18] Choi I., Cho H., Haque A.M., Bouain N., Brandizzi F., Rouached H. (2025). A Scalable Hydroponic System for Precise Nutrient and Stress Studies in Plants. Curr. Protoc..

[19] Li W., Ma L., Ye Y., Tang Q., Shen Y., Zou Z., Zhou H., Liang C., Wang G. (2025). Selenium absorption, translocation and biotransformation in pak choi (*Brassica chinensis* L.) after foliar application of selenium nanoparticles. Food Chem..

[20] Arnon D.I. (1949). Copper Enzymes in Isolated Chloroplasts. Polyphenoloxidase in Beta Vulgaris. Plant Physiol..

[21] Bradford M.M. (1976). A rapid and sensitive method for the quantitation of microgram quantities of protein utilizing the principle of protein-dye binding. Anal. Biochem.

[22] Yemm E.W., Willis A.J. (1954). The estimation of carbohydrates in plant extracts by anthrone. Biochem J..

[23] Giannopolitis C.N., Ries S.K. (1977). Superoxide Dismutases: I. Occurrence in Higher Plants. Plant Physiol..

[24] Aebi H. (1984). Catalase *in vitro*. Methods Enzymol..

[25] Heath R.L., Packer L. (1968). Photoperoxidation in isolated chloroplasts: I. Kinetics and stoichiometry of fatty acid peroxidation. Arch. Biochem Biophys..

[26] Bates L.S., Waldren R.P., Teare I.D. (1973). Rapid determination of free proline for water-stress studies. Plant Soil.

[27] (2014). National Food Safety Standard: Standard for Uses of Food Additives.

[28] Wadhwani S.A., Shedbalkar U.U., Singh R., Chopade B.A. (2016). Biogenic selenium nanoparticles: Current status and future prospects. Appl. Microbiol. Biotechnol..

[29] Abbas Q., Yousaf B., Amina, Ali M.U., Munir M.A.M., El-Naggar A., Rinklebe J., Naushad M. (2020). Transformation pathways and fate of engineered nanoparticles (ENPs) in distinct interactive environmental compartments: A review. Environ. Int..

[30] Gupta M., Gupta S. (2017). An overview of selenium uptake, metabolism, and toxicity in plants. Front. Plant Sci..

[31] Hou X., Wang Z., Peng M. (2025). Selenium compounds and their bioactivities: Molecular mechanisms and prospects for functional food and therapeutic applications. Plants.

[32] Ahmed S., Ahmad M., Swami B.L., Ikram S. (2016). A review on plants extract mediated synthesis of silver nanoparticles for antimicrobial applications: A green expertise. J. Adv. Res..

[33] Assad N., Ahmad H., Abbas A., Rehman M.F.U., Naeem-Ul-Hassan M., Sher M., Riaz T., Laila M.B., Zahra I., Ullah F. (2025). Green synthesis of selenium nanoparticles using Equisetum diffusum: Characterization, antibacterial potential, effects on plant growth. IEEE Trans. Nanobiosci..

[34] Abdelhamid A.M., Ali L.S., Fansa H.A., Srag El-Din A.S.G. (2025). Microwave-assisted synthesis of selenium nanoparticles: A comprehensive review on optimizing parameters for enhanced bioavailability and therapeutic potential. Res. J. Pharm. Technol..

[35] Shakibaie M., Khorramizadeh M.R., Faramarzi M.A., Sabzevari O., Shahverdi A.R. (2010). Biosynthesis and recovery of selenium nanoparticles and the effects on matrix metalloproteinase-2 expression. Biotechnol. Appl. Biochem..

[36] Hasanuzzaman M., Bhuyan M.H.M.B., Zulfiqar F., Raza A., Mohsin S.M., Al Mahmud J., Fujita M., Fotopoulos V. (2020). Reactive oxygen species and antioxidant defense in plants under abiotic stress: Revisiting the crucial role of a universal defense regulator. Antioxidants.

[37] Mrowiec B. (2025). Nanowaste in the aquatic environment—Threats and risk countermeasures. Desalin. Water Treat..

[38] Nile S.H., Thombre D., Shelar A., Gosavi K., Sangshetti J., Zhang W., Sieniawska E., Patil R., Kai G. (2023). Antifungal Properties of Biogenic Selenium Nanoparticles Functionalized with Nystatin for the Inhibition of *Candida albicans* Biofilm Formation. Molecules.

[39] Shahzadi S., Fatima S., Ul Ain Q., Shafiq Z., Janjua M.R.S.A. (2025). A review on green synthesis of silver nanoparticles (SNPs) using plant extracts: A multifaceted approach in photocatalysis, environmental remediation, and biomedicine. RSC Adv..

[40] Karataş H., Eker F., Akdaşçi E., Bechelany M., Karav S. (2026). Silver nanoparticles in antibacterial research: Mechanisms, applications, and emerging perspectives. Int. J. Mol. Sci..

[41] Samal P., Satpathy S., Panigrahi L.L., Jha S., Arakha M. (2025). Exploring the intricacies of protein-nanoparticle interaction and its implications in chronic diseases: A comprehensive review. Nanoscale Horiz..

[42] Tsivileva O. (2025). Selenium-containing nanoformulations capable of alleviating abiotic stress in plants. Int. J. Mol. Sci..

[43] Kumar A., Prasad K.S. (2021). Role of nano-selenium in health and environment. J. Biotechnol..

[44] Shah T., Khan Z., Alahmadi T.A., Shah M.A., Ahmad M.Z., Rasool S., Ansari M.J. (2024). Nanoselenium inhibits chromium toxicity in wheat plants by modifying the antioxidant defense system, ascorbate glutathione cycle, and glyoxalase system. Environ. Exp. Bot..

[45] Nedjimi B. (2025). The role of selenium and selenium nanoparticles in enhancing plant tolerance to cadmium stress: A sustainable approach. Discov. Plants.

[46] Singh H.P., Mahajan P., Kaur S., Batish D.R., Kohli R.K. (2013). Chromium toxicity and tolerance in plants. Environ. Chem. Lett..

[47] Samynathan R., Venkidasamy B., Ramya K., Muthuramalingam P., Shin H., Kumari P.S., Thangavel S., Sivanesan I. (2023). A recent update on the impact of nano-selenium on plant growth, metabolism, and stress tolerance. Plants.

[48] Khan Z., Thounaojam T.C., Chowdhury D., Upadhyaya H. (2023). The role of selenium and nano selenium on physiological responses in plant: A review. Plant Growth Regul..

[49] Zhang L., Chang Q., Zhao X., Guo Q., Chen S., Zhang Q., He Y., Chen S., Chen K., Ban R. (2025). Selenium improves yield and quality in Prunella vulgaris by regulating antioxidant defense, photosynthesis, growth, secondary metabolites, and gene expression under acid stress. Plants.

[50] Ali S., Mir R.A., Tyagi A., Manzar N., Kashyap A.S., Mushtaq M., Raina A., Park S., Sharma S., Mir Z.A. (2023). Chromium toxicity in plants: Signaling, mitigation, and future perspectives. Plants.

[51] Su Y., Fu F., Ou X., Gong L., Liu H., Sun Y. (2024). Response of selenium pools to drought stress by regulating physio-biochemical attributes and anatomical changes in *Gentiana macrophylla*. Ecotoxicol. Environ. Saf..

[52] Zhu S., Sun S., Zhao W., Yang X., Mao H., Sheng L., Chen Z. (2024). Utilizing transcriptomics and proteomics to unravel key genes and proteins of Oryza sativa seedlings mediated by selenium in response to cadmium stress. BMC Plant Biol..

[53] Shanker A.K., Cervantes C., Loza-Tavera H., Avudainayagam S. (2005). Chromium toxicity in plants. Environ. Int..

[54] Mansoor S., Ali A., Kour N., Bornhorst J., AlHarbi K., Rinklebe J., Abd El Moneim D., Ahmad P., Chung Y.S. (2023). Heavy metal induced oxidative stress mitigation and ROS scavenging in plants. Plants.

[55] Noor I., Sohail H., Sun J., Nawaz M.A., Li G., Hasanuzzaman M., Liu J. (2022). Heavy metal and metalloid toxicity in horticultural plants: Tolerance mechanism and remediation strategies. Chemosphere.

[56] Pei J., Pan X., Wei G., Hua Y. (2023). Research progress of glutathione peroxidase family (GPX) in redoxidation. Front. Pharmacol..

[57] Ursini F., Bosello Travain V., Cozza G., Miotto G., Roveri A., Toppo S., Maiorino M. (2022). A white paper on phospholipid hydroperoxide glutathione peroxidase (GPx4) forty years later. Free Radic. Biol. Med..

[58] Ursini F., Bindoli A. (1987). The role of selenium peroxidases in the protection against oxidative damage of membranes. Chem. Phys. Lipids.

[59] Çiğ B. (2025). Selenium reduces oxaliplatin induced neuropathic pain: Focus on TRPV1. Front. Pharmacol..

[60] Gill R.A., Zang L., Ali B., Farooq M.A., Cui P., Yang S., Ali S., Zhou W. (2015). Chromium-induced physio-chemical and ultrastructural changes in four cultivars of *Brassica napus* L.. Chemosphere.

[61] Sharma A., Kapoor D., Wang J., Shahzad B., Kumar V., Bali A.S., Jasrotia S., Zheng B., Yuan H., Yan D. (2020). Chromium bioaccumulation and its impacts on plants: An overview. Plants.

[62] Zulfiqar F., Ashraf M. (2023). Proline alleviates abiotic stress induced oxidative stress in plants. J. Plant Growth Regul..

[63] Ghosh U.K., Islam M.N., Siddiqui M.N., Cao X., Khan M.A.R. (2022). Proline, a multifaceted signalling molecule in plant responses to abiotic stress: Understanding the physiological mechanisms. Plant Biol..

[64] Shen Y., Huang H., Wang Y., Yang R., Ke X. (2022). Antioxidant effects of Se-glutathione peroxidase in alcoholic liver disease. J. Trace Elem. Med. Biol..

[65] Silva V.M., Tavanti R.F.R., Gratão P.L., Alcock T.D., dos Reis A.R. (2020). Selenate and selenite affect photosynthetic pigments and ROS scavenging through distinct mechanisms in cowpea (*Vigna unguiculata* (L.) Walp) plants. Ecotoxicol. Environ. Saf..

[66] Sharma P., Singh S.P., Tripathi R.D., Tong Y.W. (2023). Chromium toxicity and tolerance mechanisms in plants through cross-talk of secondary messengers: An overview of pathways and mechanisms. Environ. Pollut..

[67] Qin C., Lian H., Alqahtani F.M., Ahanger M.A. (2024). Chromium mediated damaging effects on growth, nitrogen metabolism and chlorophyll synthesis in tomato can be alleviated by foliar application of melatonin and jasmonic acid priming. Sci. Hortic..

[68] Liu C., Zhou G., Qin H., Guan Y., Wang T., Ni W., Xie H., Xing Y., Tian G., Lyu M. (2024). Metabolomics combined with physiology and transcriptomics reveal key metabolic pathway responses in apple plants exposure to different selenium concentrations. J. Hazard. Mater..

[69] Sun P., Ge G., Sun L., Bao J., Zhao M., Hao J., Zhang Y., Liu G., Wang Z., Jia Y. (2025). Metabolomics combined with physiology and transcriptomics reveal the regulation of key nitrogen metabolic pathways in alfalfa by foliar spraying with nano-selenium. J. Nanobiotechnol..

[70] Zhang T., Qi M., Wu Q., Xiang P., Tang D., Li Q. (2023). Recent research progress on the synthesis and biological effects of selenium nanoparticles. Front. Nutr..

[71] Xiao X., Deng H., Lin X., Ali A.S.M., Viscardi A., Guo Z., Qiao L., He Y., Han J. (2023). Selenium nanoparticles: Properties, preparation methods, and therapeutic applications. Chem. Biol. Interact..

[72] Su J., Pan X., Xian K., Fu C., He J., Liu B., Sang J., Huang N. (2025). Transcriptomic profiling of Paulownia fortunei (Seem.) Hemsl. roots in response to chromium and copper stress. Genes.

[73] Ghuge S.A., Nikalje G.C., Kadam U.S., Suprasanna P., Hong J.C. (2023). Comprehensive mechanisms of heavy metal toxicity in plants, detoxification, and remediation. J. Hazard. Mater..

[74] Yu Y., Wang Q., Wan Y., Huang Q., Li H. (2023). Transcriptome analysis reveals different mechanisms of selenite and selenate regulation of cadmium translocation in Brassica rapa. J. Hazard. Mater..

[75] Wang L.Z., Wu K.Y., Liu Z.Q., Li Z.F., Shen J., Wu Z.H., Liu H., You L.X., Yang G.D., Rensing C. (2023). Selenite reduced uptake/translocation of cadmium via regulation of assembles and interactions of pectins, hemicelluloses, lignins, callose and Casparian strips in rice roots. J. Hazard. Mater..

[76] Singh D., Sharma N.L., Singh D., Siddiqui M.H., Taunk J., Sarkar S.K., Rathore A., Singh C.K., Al-amri A.A., Alansi S. (2023). Exogenous hydrogen sulfide alleviates chromium toxicity by modulating chromium, nutrients and reactive oxygen species accumulation, and antioxidant defence system in mungbean (*Vigna radiata* L.) seedlings. Plant Physiol. Biochem..

[77] Wang Y., Feng L.J., Sun X.D., Zhang M., Duan J.L., Xiao F., Lin Y., Zhu F.P., Kong X.P., Ding Z. (2023). Incorporation of selenium derived from nanoparticles into plant proteins in vivo. ACS Nano.

[78] Wang X., Hussain B., Xin X., Zou T., Huang X., Cheng L., Wu Z., Yang Y., Li Y., He Z. (2025). Fate and physiological effects of foliar selenium nanoparticles in wheat. ACS Nano.

[79] Qu L., Xu Z., Huang W., Han D., Dang B., Ma X., Liu Y., Xu J., Jia W. (2024). Selenium-molybdenum interactions reduce chromium toxicity in Nicotiana tabacum L. by promoting chromium chelation on the cell wall. J. Hazard. Mater..

[80] Qu L., Jia W., Dai Z., Xu Z., Cai M., Huang W., Han D., Dang B., Ma X., Gao Y. (2022). Selenium and molybdenum synergistically alleviate chromium toxicity by modulating Cr uptake and subcellular distribution in *Nicotiana tabacum* L.. Ecotoxicol. Environ. Saf..

