# Supplementary material for: Nano-Selenium-Mediated Alleviation of Chromium Toxicity and Selenium Biofortification of Ipomoea aquatica Forssk. in Cyclic Hydroponic System
Source: Plants (Basel). 2026 Jul 25;15(15):2279. doi: 10.3390/plants15152279 (PMC13468092; doi:10.3390/plants15152279)
Supplement: Supplementary file 1 [file plants-15-02279-s001.zip › plants-4396884-supplementary.pdf]

Supplementary Information

Nano-Selenium-Mediated Alleviation of Chromium Toxicity and Selenium Biofortification of Ipomoea aquatica Forssk. in Cyclic Hydroponic System

Mingxuan Wang [1,2,†], Yunting Wang [3,†], Shuangqi Yue [1,2], Fengyue Qin [1,2], Menglu Dong [1,2], Wenxin Wang [1,2], Xinyu Shan [1,2], Waqas Ahmed [1,2], Sajid Mehmood [1,2,*] and Weidong Li [1,2,*]

[1] International Joint Research Center for Coral Reef Ecology of Hainan Province, School of Ecology, Hainan University, Haikou 570228, China
[2] Center for Eco-Environment Restoration of Hainan Province, School of Ecology, Hainan University, Haikou 570228, China
[3] College of the Environment & Ecology, Xiamen University, Xiamen 361102, China
* Correspondence: drsajid@hainanu.edu.cn (S.M.); weidongli@hainanu.edu.cn (W.L.)
† These authors contributed equally to this work.

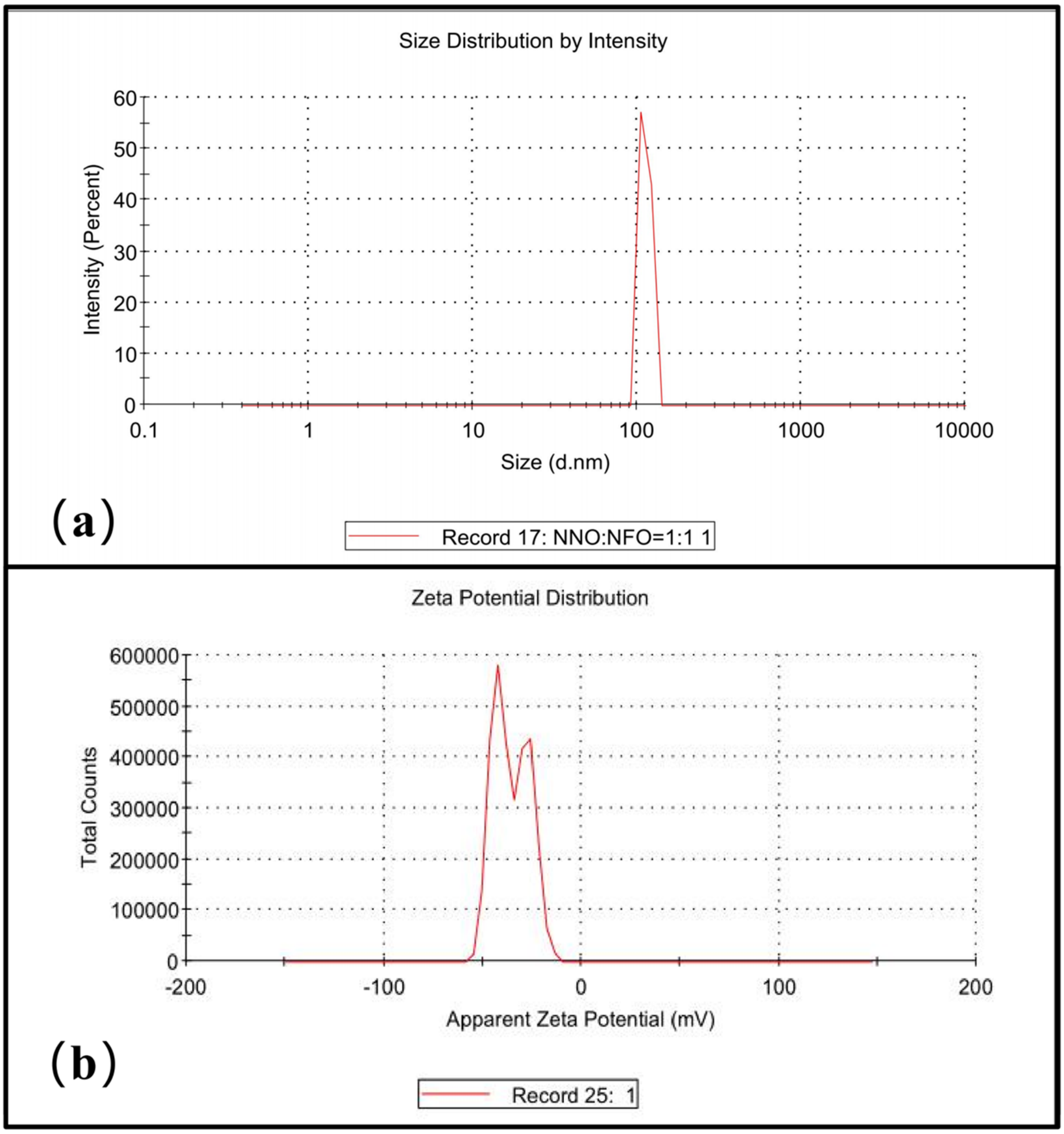

Figure S1. Physicochemical characterization of green-synthesized selenium nanoparticles (SeNPs). (a) Dynamic light scattering (DLS) size distribution, and (b) Zeta potential.

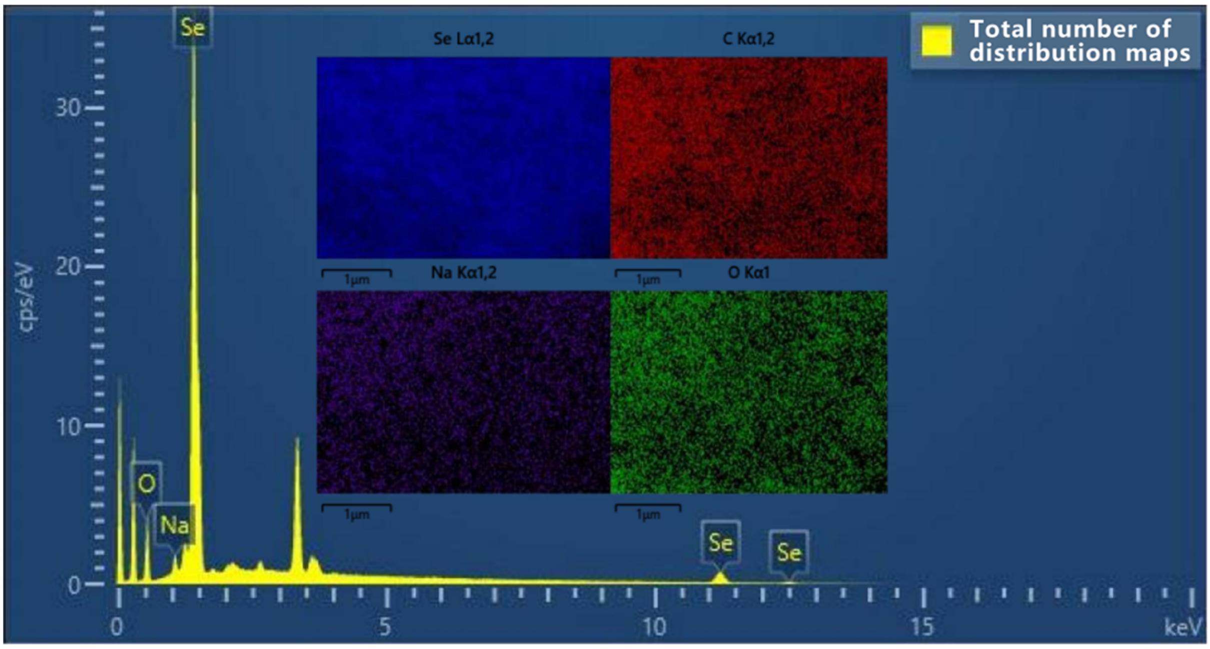

Figure S2. Elemental composition and spatial distribution of green-synthesized selenium nanoparticles (SeNPs) analyzed by energy-dispersive X-ray spectroscopy (EDS).

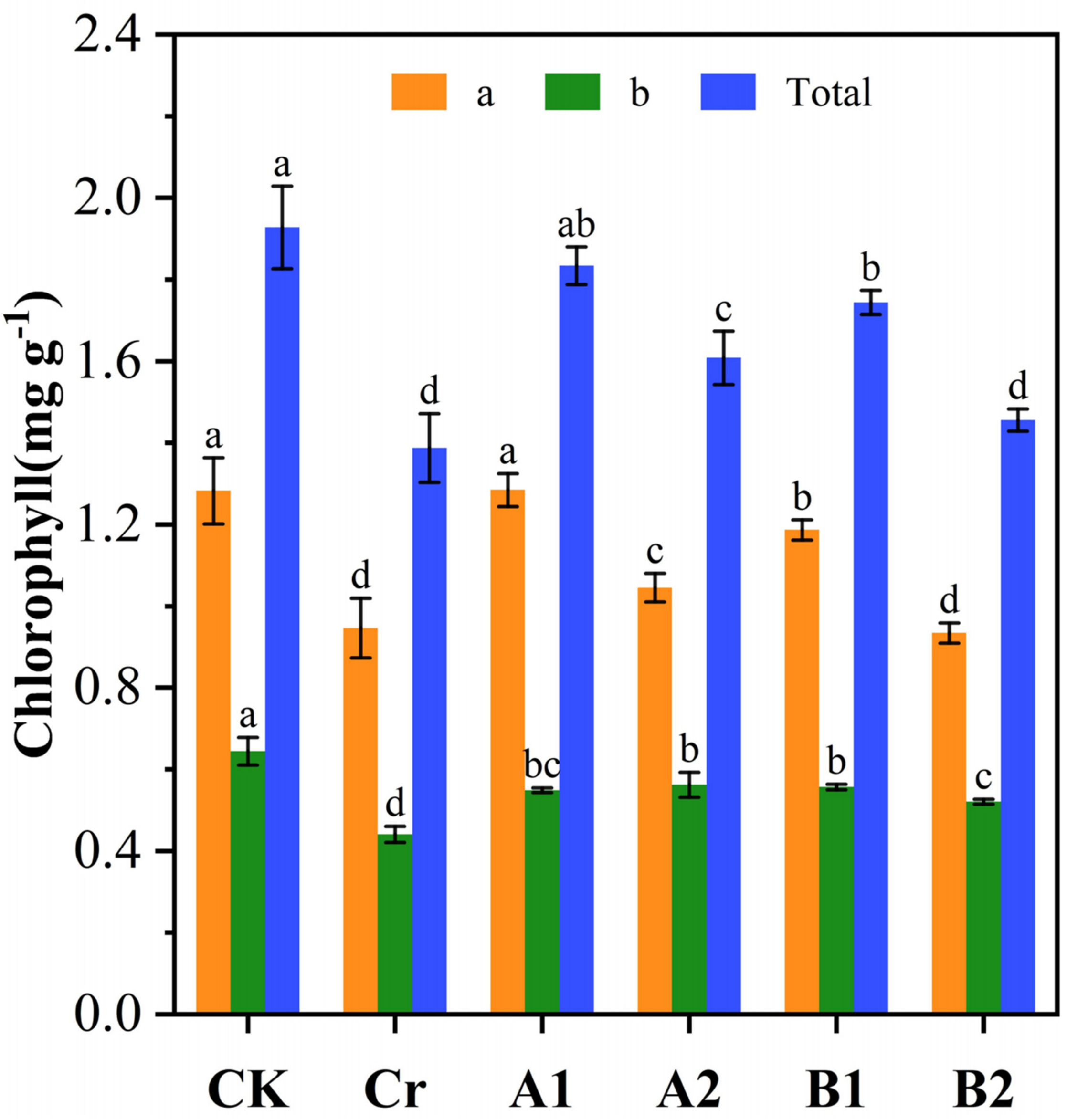

Figure S3. Effects of selenium treatments on chlorophyll content of water spinach under Cr(VI) stress. Chlorophyll a, chlorophyll b, and total chlorophyll contents were measured under different treatments.

Treatments: control (CK), Cr(VI) stress (Cr) (20 mg L^{-1}), SeNPs at 1 mg L^{-1} (A1) and 10 mg L^{-1} (A2), and Na_2SeO_3 at 1 mg L^{-1} (B1) and 10 mg L^{-1} (B2). Data are presented as mean ± standard deviation (n = 3). Different letters indicate significant differences among treatments ($p < 0.05$).

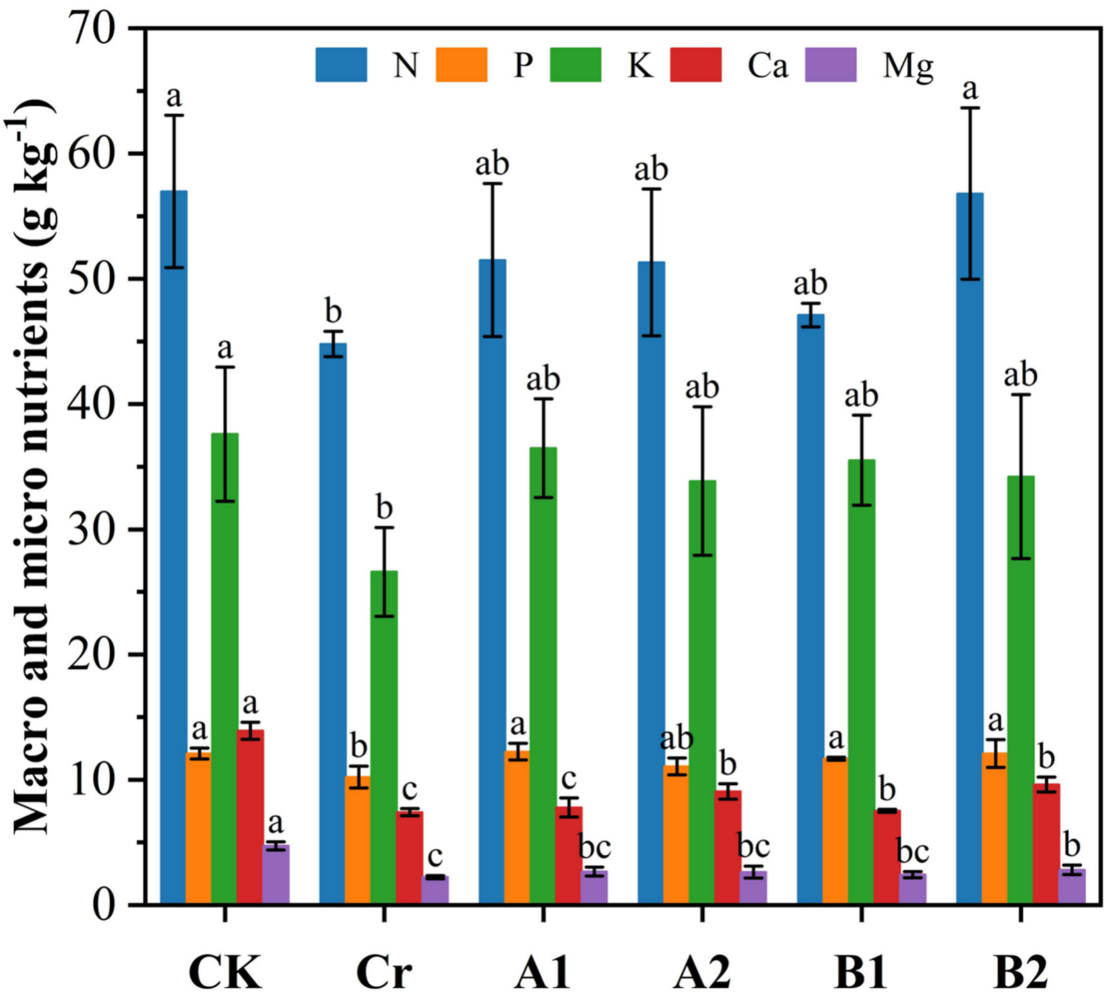

Figure S4. Effects of selenium treatments on macro- and micronutrient contents (N, P, K, Ca, and Mg) in water spinach under Cr(VI) stress.
